# Evaluation of two wild castor (*Ricinus communis* L.) accessions for cadmium tolerance in relation to antioxidant systems and lipid peroxidation

**DOI:** 10.1007/s11356-021-14844-z

**Published:** 2021-06-17

**Authors:** Akwasi Yeboah, Jiannong Lu, Shuailei Gu, Haiyan Liu, Yuzhen Shi, Hanna Amoanimaa-Dede, Kwadwo Gyapong Agyenim-Boateng, Joseph Payne, Xuegui Yin

**Affiliations:** 1 College of Coastal Agricultural Sciences, Guangdong Ocean University, Zhanjiang, China; 2 Institute of Crop Sciences, Chinese Academy of Agricultural Sciences, Beijing, China; 3 Department of Biotechnology, University for Development Studies, Tamale, Ghana

**Keywords:** Antioxidants, Cadmium, Reactive oxygen species, Tolerance index, Oxidative stress

## Abstract

The present study was conducted to assess the effect of toxicity of cadmium (Cd) on growth, tolerance index (TI), antioxidant activities, and malondialdehyde (MDA) content in two contrasting wild castor accessions (16-024 and S2-4) via hydroponic experiment (0 and 100 mg/L Cd). The results showed that Cd significantly reduced the growth rate, seedling height, root length, and shoot length of the castor accessions compared to the control, with the Cd effect being more severe in S2-4 than in 16-024. In addition, biomass response including the root and shoot fresh weight and root dry weight decreased in both accessions compared to the control. Compared to the control group, the shoot dry weight of accession S2-4 declined by 21.7%, whereas there was no change in 16-024, suggesting a level of tolerance in 16-024. Analysis of TI on all the growth parameters and biomass content revealed that accession 16-024 was highly tolerant to Cd stress than S2-4. The results further revealed that the expression of the antioxidant enzymes, viz., superoxide dismutase (SOD), catalase (CAT), non-enzymatic antioxidant, glutathione, and MDA content, was influenced by genotype. S2-4 exhibited a higher antioxidant activity (SOD, CAT) and lipid peroxidation activity than 16-024, indicative of oxidative damage from Cd stress.

## Introduction

Cadmium (Cd) is one of the highly toxic heavy metals with detrimental effects to agricultural soil, plants, and water bodies because it is highly soluble in water and readily absorbed into plant tissues (Shahid et al. 2016). Cd as a non-essential heavy metal is primarily released into environments mostly by anthropogenic or natural activities (Shahid et al. 2016). The absorption of Cd by plants negatively affects their growth, biomass, root length, and shoot length and causes leaf chlorosis and root darkening (de Souza Costa et al. 2012); however, these effects vary from plant species. Once Cd is absorbed by the plant, it enters into the human body via the food chain, thereby endangering human health (Sharma et al. 2015). Diseases such as renal tubular damage and pulmonary emphysema in humans have been associated with the consumption of Cd-contaminated foods (Sharma et al. 2015; Tinkov et al. 2018).

Cd is a non-redox active metal that does not participate in Fenton-type reactions, but it induces oxidative stress by the production of reactive oxygen species (ROS) such as superoxide anions (O_2_^●-^), hydroxyl radical (OH^●^), and hydrogen peroxide (H_2_O_2_) (Bauddh et al. 2016; Shah et al. 2001). These ROS react with lipids, nucleic acids, and proteins and cause membrane damage, enzyme inhibition, and lipid peroxidation, thus affecting the viability of cells (Gill et al. 2011). Malondialdehyde (MDA), a product of the decomposition of lipid peroxidation, is induced by oxidative stress (Kiran and Prasad 2017). Previous studies have shown that increasing Cd stress increases MDA content and is highly related to the increase in ROS levels (Bauddh and Singh 2012; Shah et al. 2001; Yu et al. 2013). Therefore, MDA is regarded as the major bio-indicator of oxidative damage in plants (Kiran and Prasad 2017). To combat the toxic effects of ROS and oxidative stress induced by Cd, plants activate ROS-detoxifying antioxidant defense mechanisms to maintain a balance between ROS production and decomposition (Gill et al. 2011; Yeboah et al. 2020a). The antioxidant defense systems consisting of enzymes such as superoxide dismutase (SOD) and catalase (CAT) and non-enzymatic antioxidant such as glutathione (GSH) help scavenge ROS to maintain the cellular redox homeostasis (Shah et al. 2001; Yeboah et al. 2020a). SOD acts as a first line of cellular defense and dismutase O_2_^●-^ to O_2_ and H_2_O_2_. CAT regulates the accumulation of H_2_O_2_ levels which later catalyzes H_2_O and O_2_ (Bauddh et al. 2016; Yu et al. 2013). GSH is a water-soluble antioxidant that directly combines with cellular electrophiles for antioxidant production or detoxification (Li et al. 2013; Shah et al. 2001). It also enhances plant responses to metal stress as a precursor of phytochelatins (PCs) and helps scavenge excess H_2_O_2_ (Zhang et al. 2014). Owing to the beneficial role of antioxidant systems in response to metal stress, several studies have been carried out on the tolerance mechanisms in diverse plant species (particularly edible plant species) under different Cd concentration and stresses (Dandan et al. 2011; Shah et al. 2001; Xu et al. 2014; Yu et al. 2013; Zeng et al. 2017) to better understand Cd tolerance mechanisms in order to and breed for tolerant accessions that can subsequently be used for phytoremediation of Cd in Cd-contaminated environments. However, information about the tolerance mechanisms of non-edible plant species such as castor to metal stress is meager.

Castor (*Ricinus communis* L.), an industrial non-edible oilseed crop belonging to the family Euphorbiaceae, is widely grown in the tropics and subtropical parts of the world. The oil in the castor oilseed is the sole oil that contains a high percentage of ricinoleic acid, making it useful for a wide spectrum of applications (Anjani 2014; Yeboah et al. 2020b). It is commercially used for medicine, agriculture, and cosmetics, among others (Anjani 2014). Aside from these, several studies have proposed its potential use as a phytoremediator (Yeboah et al. 2020c) owing to its high tolerance to heavy metal stress such as copper (Wang et al. 2016), lead (Pal et al. 2013; Prasad 2017), zinc (Wang et al. 2016), and Cd (Huang et al. 2011; Shi and Cai 2009; Ye et al. 2018; Zhang et al. 2014). From the limited studies on Cd stress, most reported that castor plants possess potential defense mechanisms, specifically the antioxidant defense systems (Bauddh et al. 2016; Bauddh and Singh 2012; Zhang et al. 2014). Nevertheless, a number of the studies were conducted in soil/field other than hydroponic culture (Bauddh et al. 2016; Bauddh and Singh 2012). Hydroponic experiment is more advantageous over soil/field experiment owing to the minimal interaction of the hydroponic medium with the experiment unlike in the soil/field where the soil and the organic matter may influence the experiment either by desorption or absorption (for metal exposure experiment) in the soil solution (de Souza Costa et al. 2012). Although hydroponic experimental conditions cannot be directly extrapolated to real field conditions, they are important as a means to determine the plant’s ability to tolerate relatively high metal concentrations maintaining fast growth rates as well as high biomass production (de Souza Costa et al. 2012).

In addition, most of the antioxidant studies in castor under Cd stress were determined at the matured stage with limited studies at the seedling stage (Bauddh et al. 2016). In reality, after sowing, the castor plant undergoes three stages, that is, the seedling stage, growth stage, and maturity stage. The matured castor plant may withstand Cd stress to some extent, whereas the seedling may not (He et al. 2020). Thus, it is imperative to evaluate the antioxidant defense systems at the seedling stage to better understand the tolerance mechanisms employed by castor against Cd toxicity and to ensure the successful selection and breeding of castor accessions with superior tolerance to Cd toxicity.

Generally, the wild castor plant has strong adaptability and resistance to various abiotic and biotic stresses compared to other materials (hybrids and cultivars) which makes them more useful for breeding purposes and remediation of contaminated soils (Kang et al. 2015). However, different wild castor accessions have different growth performance rate, adaptability, and tolerance to various abiotic and biotic stresses (Kang et al. 2015). Therefore, it is essential to ascertain the tolerance level of every accession discovered. For instance, Kang et al. (2015) reported that wild castor accessions could grow and tolerate increasing concentrations of CuSO_4_ (100 mg/L) in hydroponic culture for 20 days.

In this study, two wild castor accessions (16-024 and S2-4) obtained from the breeding unit of the Department of Crop Breeding and Genetics of Guangdong Ocean University were selected on the basis of their growth performance and responses to different Cd concentration based on our previous study (unpublished data). This study was conducted to evaluate the tolerance mechanisms and level of oxidative damage in the two wild castor accessions exposed to Cd stress in a hydroponic culture. This was achieved by evaluating the growth, tolerance index (TI), and tolerance mechanisms in the wild accessions under Cd stress. Our findings would be helpful to understand Cd mechanisms and detoxification in this plant. Also, this would be valuable for castor breeding efforts and phytoremediation in general.

## Materials and methods

### Plant material and growth condition

Seeds of two wild castor accessions 16-024 and S2-4 were provided by the Department of Crop Breeding and Genetics, Guangdong Ocean University, Zhanjiang, China. Castor seeds from each accession were surface sterilized with 2 % sodium hypochlorite solution for about 12 min and imbibed in distilled water for 24 h at 28 °C for uniform germination. The seeds were initially grown in plastic seedling trays containing vermiculite for 15 days. Uniform-sized seedlings from each accession were washed and transferred to 6-L plastic boxes containing 6 holes per box. Each box was filled with 5 L of half-strength Hoagland’s solution with the following composition: 5 μmol/L EDTAFeNa, 0.05 mmol/L NH_4_NO_3_, 0.15 μmol/L CuSO_4_, 1.9 μmol/L ZnSO_4_, 2.25 μmol/L MnCl_2_, 25 μmol/L H_3_BO_3_, 0.5 mmol/L MgSO_4_, 0.5 mmol/L KH_2_PO_4_, 2.5 μmol/L KNO_3_, and 2.5 mmol/L Ca (NO_3_)_2_ (Hoagland and Arnon 1950; Zhang et al. 2014) and left for 7 days for acclimatization. After acclimatization, six seedlings were set as control and six seedlings for Cd treatment for each accession with one hole per plant; three seedlings were used for determination of growth parameters and biochemical analyses. Cadmium chloride (CdCl_2_•2.5H_2_O, CAS: 7790-78-5) obtained from a certified company (Shanghai Macklin Biochemical Company) was used for providing Cd pollution. Twenty-two-day-old castor seedlings were exposed to Cd at 0 (control) and 100 mg/L for 5 days. We chose this concentration and number of days after conducting a trial experiment (exposure for 7 days) (unpublished data). Castor plant exposure to Cd concentration below 100 mg/L did not affect growth parameters significantly; however, Cd toxicity was observed on the root (dark root) by the fourth day, while those exposed to 100 mg/L showed a sharp decrease in growth. All the experiments were arranged in a completely randomized design and nutrient solutions were replaced every 3 days. The pH was maintained at 6.0 (± 0.1) by the addition of either 0.1 mol/L NaOH or HCl. The whole experiment took place in a controlled-climate room under strictly regulated environmental conditions at Zhanjiang Lanying Agricultural Science and Technology Co. Relative humidity was maintained between 65 and 75 %, the light/dark cycle was 16–8 h with a respective 25–27 °C temperature periodicity, and light intensity was kept at a constant 300 μmol m^−2^ s^−1^ during the daytime.

### Plant growth parameter analysis and tolerance index

Growth characteristics such as seedling height, root length, shoot length, and biomass production of both fresh weight (fwt) and dry weight (dwt) of root and shoot were measured after 5 days of Cd treatment. The seedling height, root length, and shoot length were measured using a meter scale. The fwt of biomass was measured using an electronic balance, and these same plants were later dried in an oven for 72 h at 70 °C to achieve a constant weight and were recorded as dry wt. In response to Cd stress, indices were calculated according to the following equations (Shi and Cai 2009; Wu et al. 2016).

Growth performance = [Growth parameters under Cd stress (GPcd) – Growth parameters under control (GPck)] / [GPck] × 100 % where GPcd and GPck denote root length, shoot length, and root and shoot biomass under Cd stress and CK (control), respectively.

The TI was determined based on all the growth parameters and calculated as the following (Zacchini et al. 2009): TI = [Growth parameters Cd] / [Growth parameters control] × 100 %.

### Determination of antioxidant enzyme activities

Prior to the determination of antioxidant activities, the plant leaves under stress and those without stress were harvested and immediately stored in liquid nitrogen to maintain the activity of their enzymes. About 100 mg fresh weight of leaf was homogenized with phosphate buffer solution (pH 7.4) using TissueLyser II machine for 15 min and then centrifuged at 12,000 x g for 20 min at 4 °C. The supernatants were stored at 4 °C and further used for the analysis of antioxidant enzyme activities including SOD and CAT. The SOD activity was based on the inhibition of the photochemical reduction of nitro blue tetrazolium (NBT) under H_2_O_2_ and light following the method of Beauchamp and Fridovich (1971). The reaction was initiated by illumination after riboflavin addition and terminated after 15-min incubation in the dark and the absorbance was measured at 560 nm. One SOD activity was defined as the amount of protein required to inhibit 50% initial reduction of NBT under environmental conditions and was expressed as U/g fresh weight (fw).

CAT activity was analyzed by the consumption of H_2_O_2_ at 240 nm as described by Aebi (1984). Reduction of 0.01 units at A240 per min was considered as one unit of enzyme activity (U), and CAT activity was expressed as U/g fw.

### Determination of glutathione

Approximately 100 mg fresh leaf was homogenized with phosphate buffer solution (pH = 7.4) using TissueLyser II machine and then centrifuged at 12,000 x g for 20 min at 4 °C, and the supernatants were collected. The enzyme extraction and analysis were conducted following the method described by Knörzer et al. (1996) and Eyer and Podhradský (1986).

### Determination of lipid peroxidation

The level of lipid peroxidation in the leaves was measured in terms of MDA content determined by estimating thiobarbituric acid (TBA) reactive substances as described by Dhindsa et al. (1981). Briefly, 100 mg leaf samples was homogenized in 10 mL of trichloroacetic acid (TCA, 10 %) using the TissueLyser II machine. The homogenate was centrifuged at 10,000 x g for 10 min, after which the supernatants were collected. A mixture of 1 mL of supernatant and 2 mL of 0.5% 2-TBA in 10% TCA was heated at 95 °C for 15 min and then quickly cooled in an ice bath. After centrifuging at 10,000 x g for 10 min, the absorbance of the supernatant was measured at 532 nm and corrected for non-specific turbidity by subtracting the absorbance of the same at 600 nm. The blank was 0.5% TBA in 10% TCA. The concentration of MDA (C_MDA_) in the reaction solution was calculated using the extinction coefficient (155 mM^-1^cm^-1^).

Antioxidant enzymes, GSH, and MDA were all analyzed using an ultraviolet-visible spectrophotometer (UV-1100, Metash, China) at the Department of Crop Breeding and Genetics Laboratory, Guangdong Ocean University, China.

### Statistical analysis

All data (n = 3) were analyzed using SPSS version 22.0 (SPSS, Inc., Chicago, IL). Data were subjected to paired-samples *t-*test at a confidence interval of 95% to compare the significant differences between the control and Cd-stressed plants as well as between accessions. Graphical work was done with GraphPad Prism version 8.0 (GraphPad Software, San Diego California USA, http://www.graphpad.com).

## Results and discussion

### Plant growth parameter (growth rate, seedling length, root length, and shoot length)

**Fig. 1:**
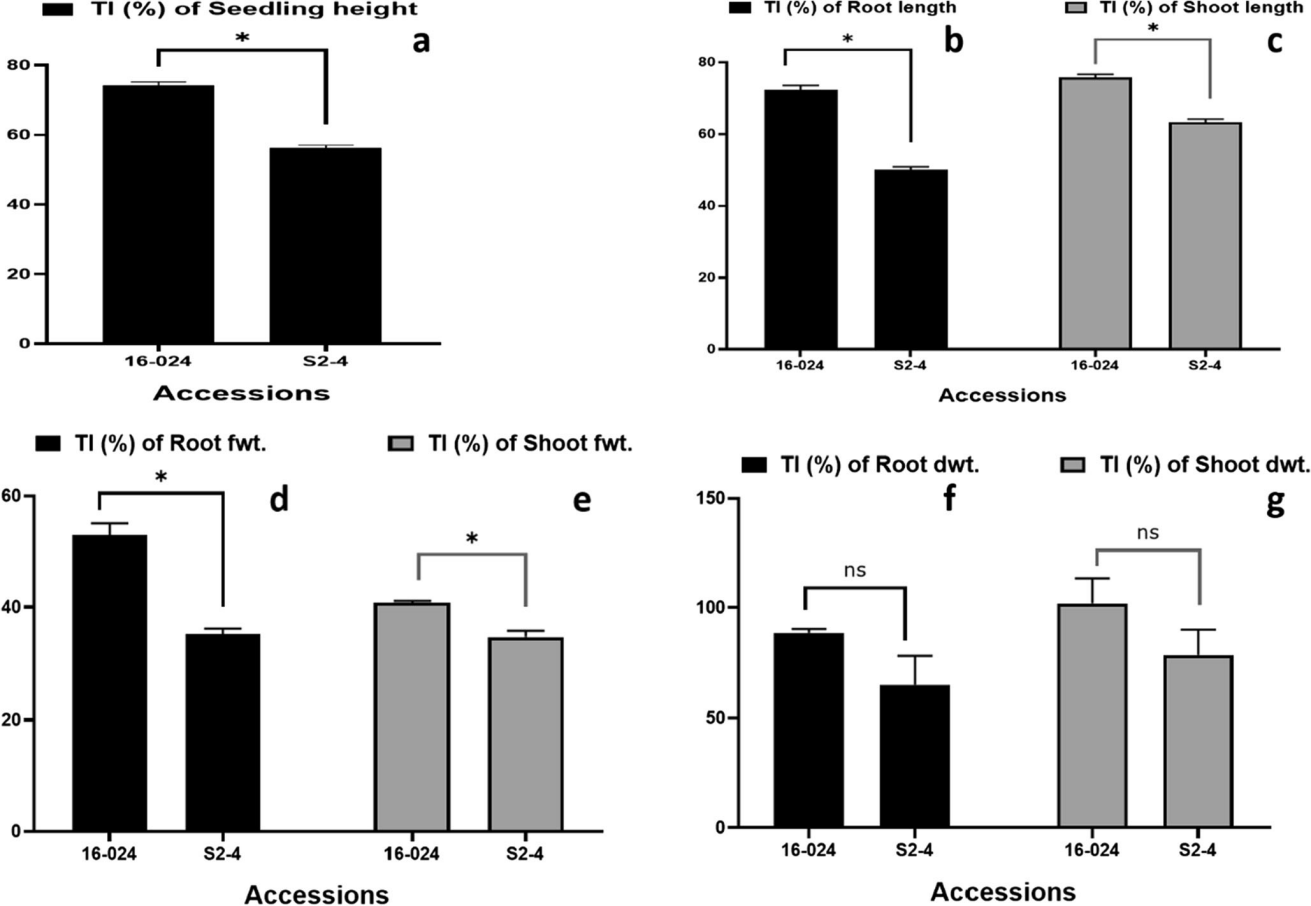
Tolerance index (TI %) of seedling height (**a**), root length (**b**), shoot length (**c**), root fwt (**d**), shoot fwt (**e**), root dwt (**f**), and shoot dwt (**g**) of castor in Cd stress (data are mean of three replicated and error bar represents standard deviation of the three replications). Paired-samples *T-*test (0.05) was performed to compare between the two wild castor accessions, where * = significant at p < 0.05 and ns = not significant

**Fig. 2:**
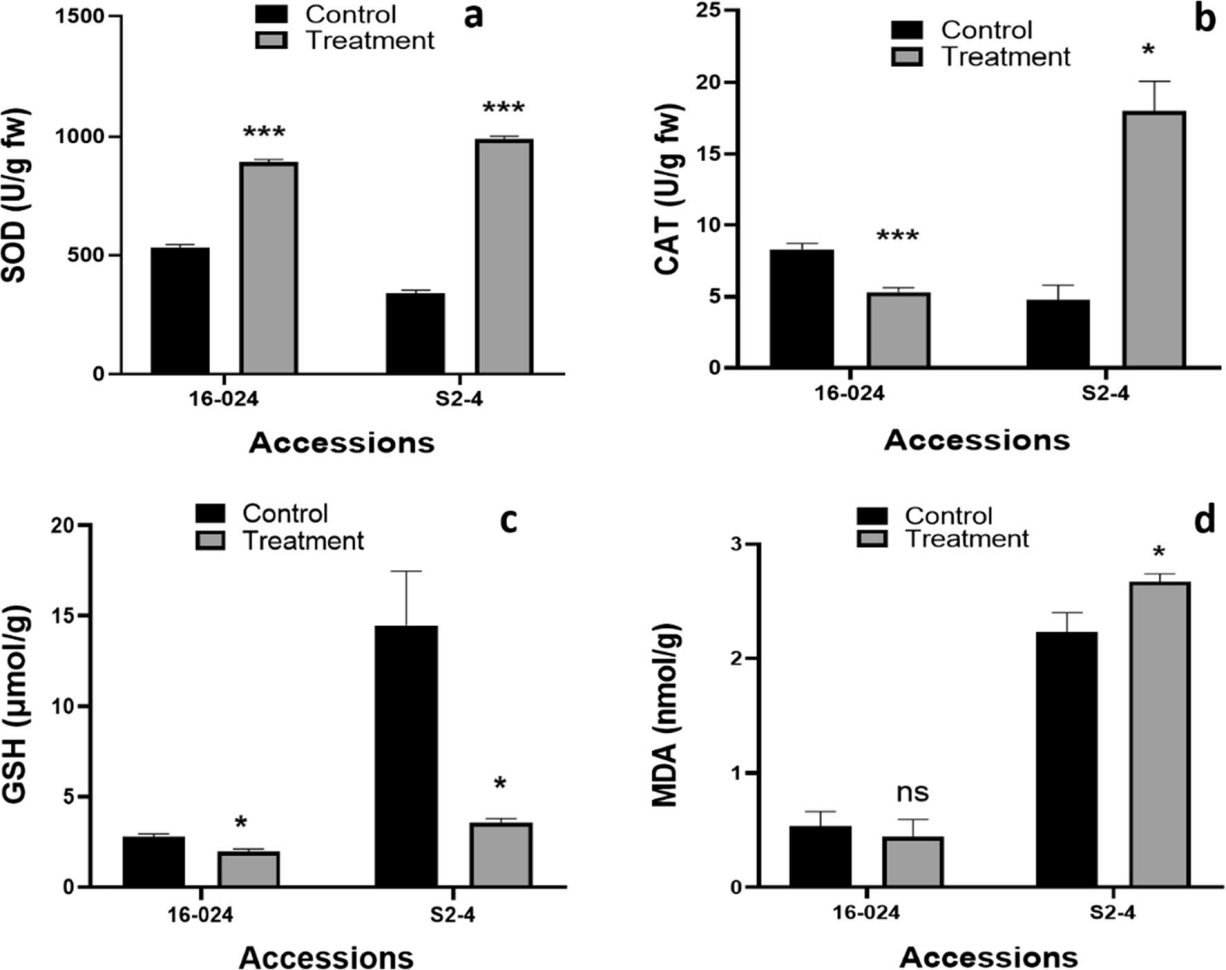
The response of superoxide dismutase (SOD, **a**), catalase (CAT, **b**) glutathione (GSH, **c**), and malondialdehyde (MDA, **d**) in leaves of two castor accessions grown under Cd stress and without stress for 5 days. Data are mean of three replicates and error bar represents standard deviation of the three replications. Significant differences from the control are indicated as * p < 0.05, *** p < 0.001, and ns as non-significant based on paired samples *T*-test (0.05)

Increased industrialization and urbanization have markedly contributed to soil contamination by Cd which impairs plant growth and development, as well as reduction of plant metabolism (de Souza Costa et al. 2012). It is established that plant accessions vary in their tolerance to Cd toxicity. Therefore, in this study, two contrasting wild castor accessions were used to investigate the growth, biomass production, TI, antioxidant systems, and lipid peroxidation in castor under Cd stress for 5 days. The results showed a wide variation between the accessions with respect to all the parameters investigated (Table 1, Figs. 1 and 2). The growth rate of the castor accessions was inhibited upon Cd treatment in the growth medium compared to their control groups and symptoms of Cd toxicity such as root darkening occurred. The inhibitory effect of Cd was more severe in S2-4 compared to 16-024. Mostly low Cd exposure (below 5 μM) results in low or no phytotoxicity depending on the plant species (Yang et al. 2018). However, high Cd concentration, especially above 100 mg/kg, has been associated with massive reduction in plant growth accompanied with high phytotoxicity such as leaf spot, chlorosis, and necrosis (Shi and Cai 2009; Yang et al. 2018). Hence, our results of Cd toxicity and reduction in growth rate observed in the castor accessions compared to the control may also be due to the high Cd concentration which restricted the intake of essential nutrients in the growth medium thereby disrupting the plant metabolism. These findings are in consonance with previous studies reported in kenaf (Li et al. 2013), maize (Xu et al. 2014), castor (Hazama et al. 2015), sugarcane (Zeng et al. 2017), and switchgrass (Guo et al. 2019). This present study confirms that high Cd concentration causes cytotoxicity and reduction in plant growth.

**Table 1:** Growth and biomass of two castor accessions under control and Cd treatment

Cd treatment (mg/L)	Seedling height (cm)	Root length (cm)	Shoot length (cm)	Fresh weight of biomass (g/plant)		Dry weight of biomass (g/plant)	
				Root	Shoot	Root	Shoot
16-024							
0	36.2 ± 2.13	16.0 ± 1.00	20.2 ± 1.13	3.18 ± 0.12	8.61 ± 0.15	0.09 ± 0.01	0.15 ± 0.01
100	26.8 ± 1.23*	11.6 ± 0.54*	15.3 ± 0.70*	1.68 ± 0.08*	3.51 ± 0.03*	0.08 ± 0.01*	0.15 ± 0.02^ns^
*t* value	17.93	16.51	19.32	24.46	73.61	17.32	− 0.31
	(*p =* 0.003)	(*p =* 0.004)	(*p =* 0.003)	(*p =* 0.002)	(*p =* 0.001)	(*p =* 0.003)	(*p =* 0.787)
S2-4							
0	36.2 ± 1.43	19.3 ± 0.76	16.8 ± 0.67	2.50 ± 0.04	5.45 ± 0.13	0.08 ± 0.006	0.15 ± 0.004
100	20.3 ± 1.11*	9.7 ± 0.54*	10.7 ± 0.57*	0.88 ± 0.02*	1.89 ± 0.02*	0.05 ± 0.006*	0.12 ± 0.012*
*t* value	85.35	75.43	106.63	69.29	43.71	5.19	6.03
	(*p =* 0.001)	(*p =* 0.001)	(*p =* 0.001)	(*p =* 0.001)	(*p =* 0.001)	(*p =* 0.035)	(*p =* 0.026)

Data are mean of three replicates ± standard deviation. Paired-samples *T-*test (0.05) was performed to compare the means of control and treatment, where * = significant and ns = not significant

The seedling height, root length, and shoot length of the castor accessions in Cd stress decreased compared to their controls (Table 1). Cd stress decreased seedling height of plants by 25.8% in 16-024 and 43.8% in S2-4 with respect to their control group. Compared to the control, the declines in root and shoot length of 16-024 were 27.7% and 24.3%, respectively; the decreased values of root and shoot length of S2-4 were 50.0% and 36.6%, suggesting a higher decrease in S2-4 than in 16-024, respectively. A possible explanation for the higher decrease in height, root length, and shoot length in S2-4 may be due to its higher sensitivity to Cd stress. Also, a similar study has reported a wide variation between two kenaf accessions when exposed to 20 μ/mol Cd in the growth medium. It was found that the kenaf Cd-sensitive cultivar ‘ZM412’ recorded a higher decrease in root and shoot length compared to the Cd-tolerant cultivar ‘Fuhong 991’ (Li et al. 2013). Yang et al. (2018) reported a significant reduction in length which varied among seven genotypic *Salix* species upon Cd exposure.

### Biomass response under Cd stress

Biomass reduction is a general response of higher plants and an important indicator to evaluate metal toxicity in plants. Previous studies have reported a decline in root and shoot biomass due to Cd stress (de Souza Costa et al. 2012; Shi and Cai 2009; Xu et al. 2014; Zeng et al. 2017). Table 1 shows that the fwt and dwt of biomass of the castor accession under Cd stress decreased compared to their control groups. Suppression of fwt of root and shoot biomass in 16-024 were 47.1% and 59.2%, respectively, and those of S2-4 were 64.8% and 65.3%, compared to their control groups. Compared to the control group, the root dwt of 16-024 significantly decreased (*p <* 0.05) by 11.5% whereas there was no statistical difference in shoot dwt, suggesting certain level of tolerance in this accession to Cd stress. Conversely, S2-4 had both root and shoot dwt decreased (*p <* 0.05) by 36.0% and 21.7%, respectively, compared to the control groups (Table 1). The biomass variation between the two castor accessions in response to Cd stress could be attributed to the differences in their genetic makeup. 16-024 was relatively tolerant to Cd stress and exhibited a minimal reduction in all growth parameters and biomass production compared to S2-4 in the growth medium. Nevertheless, to better understand and distinguish the tolerance ability between the two castor accessions, the TI and biochemical mechanisms were further investigated in this study.

### Tolerance index

Tolerance index is a useful indicator to assess plant tolerance under metal stress. Indexes with high value signify higher tolerance and indexes with low value indicates lower tolerance or higher metal effect on plant (Shi and Cai 2009). The TI of seedling height, root length, shoot length, root dwt, shoot dwt, root fwt, and shoot fwt are shown in Fig. 1. Comparatively, the TI of seedling height of 16-024 significantly increased (*p* < 0.05) by 74.2% and S2-4 by 56.2% (Fig. 1a). Also, the TI of root and shoot length of 16-024 were 72.3% and 75.7%, respectively. Those values were significantly higher than the root length (*p* < 0.05) and shoot length (*p* < 0.05) of S2-4 which increased by 50.0% and 63.3%, respectively. The root and shoot fwt of 16-024 significantly increased by 52.9% and 40.8%, respectively, compared to the root fwt and shoot fwt of S2-4 which were 35.2% and 34.7%. The TI of root dwt of 16-024 was higher (88.4%) but not significant (*p* > 0.05) compared to the root dwt of S2-4 (64.8%). Similarly, the paired-samples *T*-test showed that there was no significant difference (*p* > 0.05) between the shoot dwt of 16-024 (101.9%) and S2-4 (78.4%). The higher tolerance of 16-024 under Cd stress is attributed to the higher values compared to S2-4. Recently, Palanivel et al. (2020) reported high TI of castor plant height (48–133%), root dry wt. (25–360%), and shoots dry wt. (18–328%) when grown in five different types of copper mined soils and slag. The high TI value reported in their study compared to this present study may be associated with differences in metals and experimental conditions.

### Antioxidative enzymatic activities

To combat Cd-induced oxidative damage, the plant’s cell and organelle employ different antioxidant enzymes that help to control redox potential and tolerate oxidative stress. The production of antioxidant enzymes including SOD and CAT, among others, plays important roles to sequester, neutralize, or detoxify Cd toxicity as a result of ROS production, thereby enhancing plant tolerance (Singh et al. 2016; Yeboah et al. 2020c). SOD is a metalloprotein that catalyzes the dismutation of two molecules of O^2-^ to O_2_ and H_2_O_2_ and helps protect cells against the toxic effects of O^2-^ produced in different cellular components (Bhaduri and Fulekar 2012) and CAT is a primary enzyme that regulates intracellular H_2_O_2_ level and catalyzes into H_2_O and divalent oxygen (Bhaduri and Fulekar 2012). The activities of SOD and CAT in the two castor accessions under Cd stress and their control groups are shown in Fig. 2a and b. The activity of CAT significantly decreased (*p* < 0.001) in 16-024 and increased (*p* < 0.05) in S2-4 compared to their control group. SOD and its activity significantly increased (*p* < 0.001) in the castor accessions under Cd treatment compared to their control groups. Increases in SOD and CAT activities were higher in S2-4 (1002.0 ± 12.20 U/g fw and 18.97 ± 2.40 U/g fw) than in 16-024 (891.9 ± 11.47 U/g fw and 5.30 ± 0.32 U/g fw). The higher SOD and CAT activities in S2-4 may be explained by the higher production of ROS in the leaves which was effectively converted to H_2_O_2_ by SOD and further converted to reusable by-products (water and oxygen molecules) by CAT. On the other hand, the decrease in CAT activity in 16-024 may be due to high reactive singlet molecular oxygen which inhibited the enzyme activity or disruption of protein synthesis caused by Cd toxicity (Ma et al. 2017). Another possible reason for the decrease in CAT may be due to the fact that the enhanced levels of SOD could not be sufficient to remove completely the generated ROS, which are capable of provoking the inhibition of CAT. The better coordination of enzymatic activities as witnessed by the maximum (S2-4) increases in SOD and CAT in the leaves of the S2-4 castor accession suggest effectiveness as the first line of defense in scavenging ROS and function in Cd detoxification in the leaves of castor plant. In agreement with our study, Zeng et al. (2 017) reported elevated levels of SOD activities (30.31–150.15%) in the leaves of sugarcane among five different accessions which effectively eliminated oxidative stress caused by Cd toxicity. Existing studies have also reported enhanced SOD activities upon Cd stress (He et al. 2013; Li et al. 2013) except for Xu et al. (2014) who reported a decline in SOD activity in maize seedlings which was attributed to inhibition caused by increasing H_2_O_2_ in the Cd treatment. Similarly, increase and decrease in CAT activity have been reported in rice (Yu et al. 2013), poplar (He et al. 2013), castor (Zhang et al. 2015), and sugarcane (Zeng et al. 2017) upon Cd exposure in the growth medium, which can be attributed to the different plant species and tissues, or concentrations and durations of metal exposure (Zhang et al. 2015). The different responses of antioxidant enzymes (SOD and CAT) in our study to Cd seem to be genotype-specific.

### Non-antioxidant enzymes

GSH is a key component of non-protein thiols responsible for maintaining the cellular redox status. GSH acts as a chelating bio-ligand that neutralizes the oxidative stress caused by metal toxicity in plants. It also plays an important role by scavenging excess H_2_O_2_ and reacts with hydrogen radical, superoxide radical, and singlet oxygen (Cobbett 2000; Hall 2002). Increasing GSH levels is important for Cd detoxification since it is a precursor of PCs (components of non-protein thiols), which form complexes with Cd and sequester it into the vacuoles (Cobbett 2000). However, the present results revealed a concomitant decrease of GSH level in both accessions (Fig. 2c), with the effect being more severe in S2-4 (3.54 ± 0.24 μmol/g) than 16-024 (2.0 ± 0.14 μmol/g) which may be due to the increase of PC leading to higher resistance of plants to oxidative stress (Zhang et al. 2014). This finding conforms with that reported in castor (Zhang et al. 2014), *Brassica chinensis* L (Lou et al. 2017), and *Zea mays* L. (Singh et al. 2019), where decreased GSH levels under Cd stress were attributed to PC synthesis. Thus, it may be deduced that the depletion of GSH to reduce the toxicity of Cd could be a primary mechanism for plants to adapt to Cd stress.

### Lipid peroxidation

MDA is the cytotoxic product of membrane lipid peroxidation, and it accumulates when plants are exposed to oxidative stress. Therefore, the MDA content is mostly considered as a basic biomarker of lipid peroxidation and the stress level (Guo et al. 2014). In this study, we found that accumulation of MDA was not statistically different (*p* > 0.05) in accession 16-024 and significantly increased (*p* < 0.05) by 2.67 ± 0.07 nmol/g in accession S2-4, upon exposure to Cd as compared to their controls (Fig. 2d). This result is consistent with that reported in two different rice cultivars, where MDA content was higher in Cd-sensitive cultivar ‘Xiushui 10’ than in Cd-tolerant cultivar ‘Xiushui 11’ when treated with Cd (0, 100, 200, and 400 μM) stress for 3 days in the growth medium (Wang et al. 2015). Bauddh and Singh et al. (2016) also reported higher MDA contents in the leaves (4.53-fold ) of *Brassica juncea* L. compared to the leaves (2.8-fold) of castor when subjected to Cd treatment. MDA levels reflect the degree of cell membrane damage caused by oxidative stress. Hence, the increased MDA content of Cd-sensitive accession S2-4 indirectly explains the oxidative stress induced by Cd stress compared with that of 16-024. Conversely in 16-024, the similarity in MDA content between the control and treatment group proposes the lack of free radical accumulation upon exposure to Cd stress, hence the observed tolerance of this accession. The antioxidant enzymes particularly SOD and CAT act as the first line of defense against free radicals, and higher SOD activities in different cellular compartments under Cd stress signify higher O^2-^, capable of causing oxidative damage (Zhang et al. 2015). Therefore, in this study, the higher decrease in the growth parameters and increase in MDA content and antioxidant enzymes in accession S2-4 may explain its sensitivity to Cd stress compared to 16-024.

## Conclusion

This study was undertaken to comparatively evaluate two contrasting wild castor accessions to Cd stress. From this study, it can be concluded that the growth and tolerance of castor under Cd toxicity vary between accessions. The addition of Cd in the growth medium inhibited the growth and biomass of castor and the level of tolerance to Cd toxicity varied between the accessions. Accession S2-4 was highly sensitive to Cd toxicity which was observed in the lower TI, increase in MDA content, and antioxidant enzymes compared to 16-024. Although the expression of the antioxidant enzymes in 16-024 were low compared to S2-4, it may be that Cd stress did not induce much ROS production as observed by the similar MDA content compared to control, and hence, the antioxidant enzymes did not have much ROS to scavenge. This renders 16-024 as a more tolerant accession and better suited for further screening and utilization in genetic breeding improvement programs aimed at Cd tolerance over S2-4. However, it is recommended that future studies should include multi-omics strategies (transcriptomics and metabolomics) to unravel transcript and metabolite regulation in these two accessions to boost our understanding.

## Data Availability

The datasets used and/or analyzed during the current study are available from the corresponding author on reasonable request.
